# Genome sequencing identifies an attenuated, cardiac-predominant form of mucolipidosis type IIIα/β as the potential cause of a genetically elusive, ring-like arrhythmogenic non-dilated left ventricular cardiomyopathy

**DOI:** 10.1016/j.hrcr.2026.06.016

**Published:** 2026-06-22

**Authors:** Matteo Castrichini, Evelyn T. Kim, David J. Tester, Lisa A. Schimmenti, Michael J. Ackerman, John R. Giudicessi

**Affiliations:** 1 Department of Cardiovascular Medicine, Mayo Clinic, Rochester, Minnesota; 2 Department of Molecular Pharmacology and Experimental Therapeutics, (Windland Smith Rice Sudden Death Genomics Laboratory), Mayo Clinic, Rochester, Minnesota; 3 Departments of Genetics, Mayo Clinic, Rochester, Minnesota; 4 Department of Pediatrics (Division of Pediatric Cardiology), Rochester, Minnesota

**Keywords:** Whole genome sequencing, Mucolipidosis, Arrhythmogenic Cardiomyopathy, Late gadolinium enhancement, Cardiac magnetic resonance

**Key Teaching Points**

• Variants in *GNPTAB*, typically associated with mucolipidosis type IIIα/β, can present with isolated or predominant cardiac involvement, mimicking primary arrhythmogenic cardiomyopathy despite only subtle extra-cardiac features.
• Genome sequencing can uncover rare, actionable etiologies in cardiomyopathy cases that are negative on standard gene panels, as demonstrated by the identification of compound heterozygous *GNPTAB* variants in a patient with arrhythmogenic left ventricular cardiomyopathy.
• The ring-like pattern of subepicardial late gadolinium enhancement on cardiac magnetic resonance imaging is a distinctive imaging feature that may point toward nontraditional causes of arrhythmogenic left ventricular cardiomyopathy, including lysosomal storage disorders.

## Introduction

Arrhythmogenic cardiomyopathy (ACM) encompasses a spectrum of genetically driven myocardial diseases characterized by fibrofatty replacement of the ventricular myocardium and a predisposition to life-threatening arrhythmias. Although arrhythmogenic right ventricular cardiomyopathy is traditionally associated with desmosomal gene variants, increasingly diverse genetic substrates and phenotypic presentations are being recognized, especially in the left-sided forms. We present the case of a 38-year-old man with a cardiac-predominant arrhythmogenic left ventricular cardiomyopathy phenotype, ultimately diagnosed through genome sequencing (GS) with a syndromic lysosomal storage disorder. This case highlights the diagnostic value of comprehensive genomic testing in patients with unexplained cardiomyopathy and underscores the limitations of conventional gene panels.

## Case report

**Figure 1:**
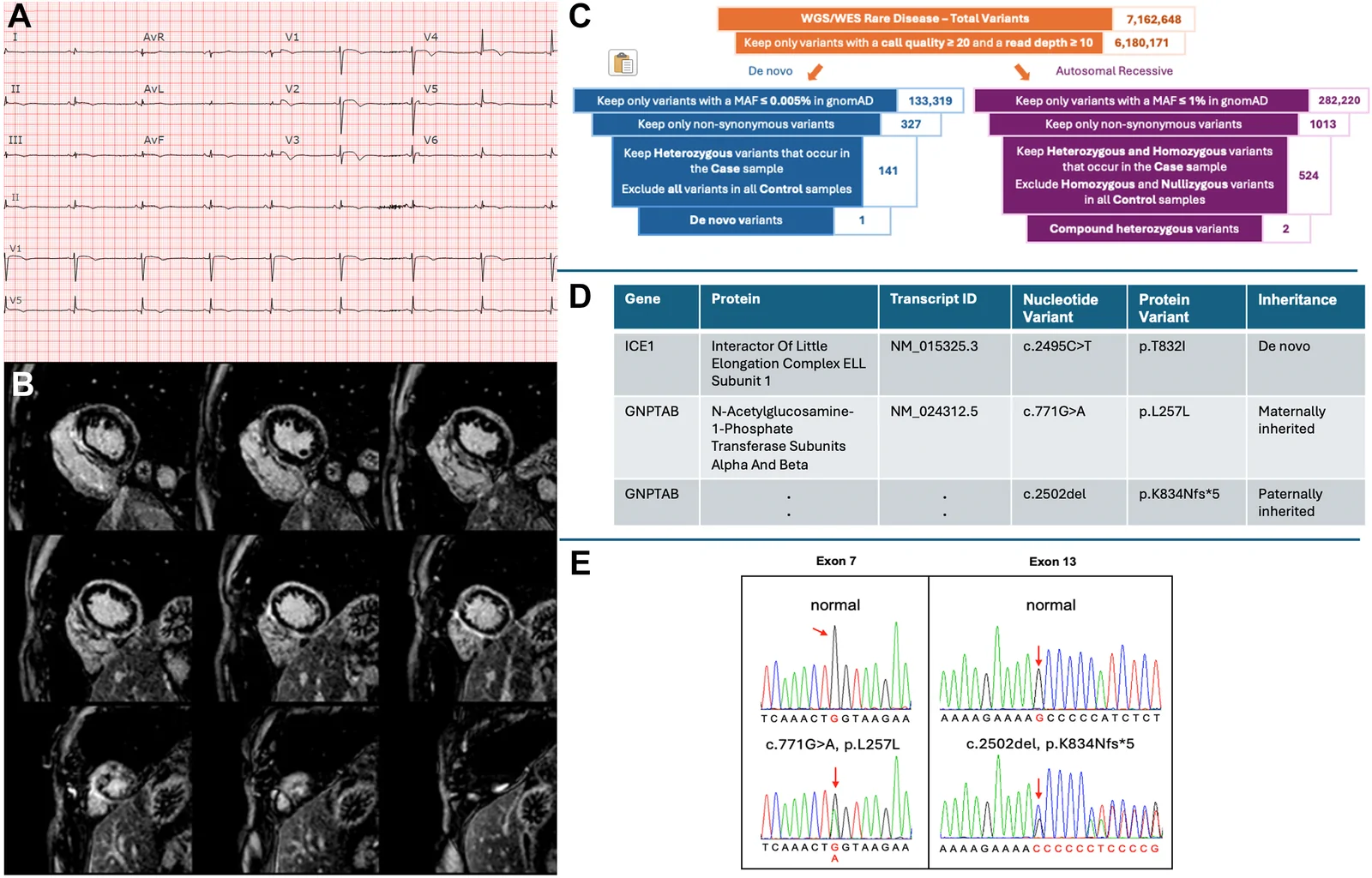
Genome sequencing identifies an attenuated, cardiac-predominant form of mucolipidosis type IIIα/β as the potential cause of a genetically elusive, ring-like arrhythmogenic non-dilated cardiomyopathy. **A**: A 12-lead electrocardiogram showing extensive T-wave inversion in the inferolateral and anterior leads and ST elevation in the precordial leads. **B**: Postcontrast cardiac magnetic resonance imaging short-axis images showing extensive subepicardial delayed enhancement in a ring-like pattern. **C**: Schematic representation of the variant filtering strategy used to identify ultra-rare non-synonymous candidate variants present in the affected and non-affected individuals who underwent genome sequencing. **D**: The 3 ultra-rare variants identified by genome sequencing that were considered for further analysis based on the literature review. Based on biological plausibility, the compound heterozygous *GNPTAB* variants represented the top candidate disease-causing variants. **E**: Sanger sequencing confirmation of the compound heterozygous c.771G>A (p.Leu257Leu) and c.2502del (p.lys834Asnfs∗5) *GNPTAB* variants.

A 38-year-old man with no personal prior medical history presented to his local emergency department with chest pain and palpitations. Initial evaluation revealed normal troponin levels, low voltage with extensive T-wave inversions on 12-lead electrocardiogram (Figure 1A), and frequent premature ventricular contractions. A detailed cardiac workup, including normal coronary angiography, was performed. Transthoracic echocardiogram showed reduced left ventricular (LV) systolic function with regional wall motion abnormalities, including apical hypokinesis. Cardiac magnetic resonance (CMR) revealed normal LV size and wall thickness, dyskinesis of the LV apex, an LV ejection fraction of 34%, and extensive near-circumferential “ring-like” subepicardial late gadolinium enhancement (LGE) without edema (Figure 1B). Collectively, these electrocardiographic and imaging findings met diagnostic Padua criteria for arrhythmogenic left ventricular cardiomyopathy. Given the extensive myocardial fibrosis at CMR and high arrhythmic burden on ambulatory monitoring, the patient underwent implantable cardioverter defibrillator (ICD) placement for primary prevention. He was referred to the Mayo Clinic Windland Smith Rice Sudden Death Genomics Laboratory after a negative commercially available pan-cardiac (n = 168 genes) genetic testing.

GS was performed on DNA from the patient and his unaffected parents. Variants were filtered based on inheritance patterns (sporadic de novo or autosomal recessive) using dedicated software (Figure 1C). Variants were first filtered for call quality (≥20) and read depth (≥10). Non-synonymous variants (eg, missense, nonsense, frameshift, splice errors) identified in the proband were prioritized. For the de novo model, ultra-rare variants (minor allele frequency [MAF] ≤ 0.005% in gnomAD)^1^ absent in both parents were considered. For the recessive model, rare variants (MAF ≤ 1%)^1^ present as homozygous or compound heterozygous (2 unique variants, each inherited from a different parent) were evaluated. These thresholds were determined using an ACM prevalence of 1 in 2,000 and assuming 50% penetrance with 1% maximum allelic contribution. Candidate variants were further prioritized using combined annotation-dependent depletion scores and the Human Protein Atlas (https://www.proteinatlas.org/). Variants with combined annotation-dependent depletion scores of ≥10 and cardiac protein expression were included. After GS and inheritance modeling, 3 candidate variants in 2 genes (*ICE1* and *GNPTAB*) were identified (Figure 1D). Compound heterozygous variants in *GNPTAB*, encoding N-acetylglucosamine-1-phosphate transferase (GlcNAc-phosphotransferase), were confirmed using Sanger sequencing. These included a maternally inherited splice-altering variant (c.771G>A, p.Leu257Leu) in exon 7 and a paternally inherited frameshift mutation (c.2502del, p.Lys834Asnfs∗5) (Figure 1E). Follow-up genetic counseling and metabolic testing revealed normal urinary and blood biomarkers for lysosomal storage disorders, normal enzyme activity, and unremarkable skeletal findings. The patient exhibited normal cognition, only subtle coarse facial features, and interestingly mild corneal clouding and cataracts.

Shortly after ICD placement, the patient experienced an asymptomatic episode of ventricular tachycardia, terminated by anti-tachycardia pacing. He was subsequently hospitalized in an outside facility following a ventricular tachycardia storm, accompanied by syncope, requiring multiple ICD shocks and initiation of amiodarone therapy.

## Discussion

Biallelic pathogenic variants in *GNPTAB* lead to GlcNAc-phosphotransferase deficiency, impairing mannose-6-phosphate tagging of lysosomal enzymes. This deficiency causes mucolipidosis type IIIα/β (pseudo-Hurler polydystrophy), a rare autosomal recessive lysosomal storage disorder.^2^ Although commonly presenting in childhood with features such as short stature, joint stiffness, mild intellectual disability, and corneal clouding, this condition also involves cardiac abnormalities, including valve disease and, less frequently, cardiomyopathy.^2^ Our patient presented with a predominant cardiac phenotype with a non-dilated left-sided cardiomyopathy with extensive subepicardial myocardial fibrosis in a ring-like pattern. Interestingly, the patient’s p.Leu257Leu (c.771G>A) variant has been previously reported as the cause of an adult-onset form of Mucolipidosis IIIα/β associated with cardiomyopathy and sensory neuropathy.^3^ Although considered a milder form of mucolipidosis type II, severe cardiac manifestations, including sudden cardiac death, have been documented.^4^ Some cases demonstrate biventricular dilated cardiomyopathy with arrhythmogenic features and diffuse LGE on CMR, similar to our patient.^5^

This case highlights a potential genetic substrate for ACM with a unique ring-like LGE pattern, commonly seen in patients with disease-causative variants in *DSP* and *FLNC*. GS analysis identified compound heterozygous *GNPTAB* pathogenic variants causing an attenuated, cardiac-predominant form of mucolipidosis type IIIα/β with mild syndromic features. Importantly, these findings underscore the limitations of traditional pan-cardiac gene panels, which failed to detect the causative variants because *GNPTAB* was not included.

## Conclusion

This case highlights the role of GS in identifying rare, actionable genetic etiologies in undifferentiated cardiomyopathies presumed “genotype-negative” using standard approaches. Given the potential for cardiac-predominant presentations, *GNPTAB* should be included in pan-cardiomyopathy genetic panels. Moreover, our findings demonstrate the value of GS in uncovering rare genetic diagnoses and guiding personalized care for complex cardiomyopathies.

## Funding sources

This work was supported by the Mayo Clinic Windland Smith Rice Comprehensive Sudden Cardiac Death Program.

## Disclosures

Dr Ackerman is a consultant for Abbott, ARMGO Pharma, Boston Scientific, Bristol Myers Squibb, Illumina, Invitae, Medtronic, Tenaya Therapeutics, and UpToDate. Dr Ackerman and Mayo Clinic are involved in an equity/royalty relationship with AliveCor, Anumana, Prolaio, Solid Biosciences, and Thryv Therapeutics. Dr Giudicessi is a consultant for Atrium Biosciences, CKD USA, Inc, Citizen Health and Nuevocor Therapeutics. In addition, Dr Giudicessi is the principal investigator for clinical trials sponsored by Nuevocor Therapeutics, Rocket Pharmaceuticals, Solid Biosciences, Tenaya Therapeutics and Thryv Therapeutics. The rest of authors have no conflicts of interest.
